# Camera-enabled techniques for organic synthesis

**DOI:** 10.3762/bjoc.9.118

**Published:** 2013-05-31

**Authors:** Steven V Ley, Richard J Ingham, Matthew O’Brien, Duncan L Browne

**Affiliations:** 1 Department of Chemistry, University of Cambridge, Lensfield Road, Cambridge CB2 1EW, UK; 2 Lennard-Jones Laboratories, School of Physical and Geographical Sciences, Keele University, Staffordshire ST5 5BG, UK

**Keywords:** automation, computer vision, digital camera, flow chemistry, machine-assisted synthesis

## Abstract

A great deal of time is spent within synthetic chemistry laboratories on non-value-adding activities such as sample preparation and work-up operations, and labour intensive activities such as extended periods of continued data collection. Using digital cameras connected to computer vision algorithms, camera-enabled apparatus can perform some of these processes in an automated fashion, allowing skilled chemists to spend their time more productively. In this review we describe recent advances in this field of chemical synthesis and discuss how they will lead to advanced synthesis laboratories of the future.

## Introduction

The increasing prevalence of digital camera technology for capturing images, videos and visible information is having a profound impact on many aspects of our modern society. Whether simply used to produce a family archive of digital photographs and videos, or in a scientific setting to enable remote interaction with exploration robots searching for evidence of life on Mars [1] or submarines charting deep ocean trenches, the current rapid growth in technological advancement (and reduction in cost) is providing us with a wealth of new enabling tools and opportunities. Recent developments include vehicles that can perform complex manoeuvres automatically [2], real-time facial recognition from security camera images [3], and camera-based pulse and respiratory rate monitoring [4–5]. When used as an aid to teaching [6–8] or to record scientific experiments [9–10], digital imagery and video-clips play a pivotal role in disseminating information. Most scientific publication houses now readily accommodate coloured imagery and video attachments [11–13] to enhance research manuscripts.

Cameras are only a part of this revolution; touch-screen devices, voice recognition, barcode and radio frequency (RF) tagging, geolocation and cloud computing are all helping to drive a rapidly changing landscape. Digital imagery is one of the cornerstones of modern communication technology, and embracing the digitisation of images and other scientific data can bring great changes to the laboratory environment; for instance, the increasing popularity of electronic laboratory notebooks (ELNs) is enabling collaborative work to take place openly and in real time [14–15]. In our vision for a “Lab of the Future” [16–18] we anticipate further integration of a multitude of wirelessly connected devices such as environmental sensors, tablet computers for displaying and recording data, and novel information aids such as safety goggles incorporating head-up displays, which might be based on recent developments in this area, such as the Google Glass project [19]. These could provide pertinent data about the procedure in hand, or alert the chemist to developing hazards elsewhere in the laboratory. The development of mobile chemistry applications (Apps) is now widespread and growing at a phenomenal rate [20] giving the modern researcher instant access to a huge variety of information. Futuristic laboratories may be built around smart fume hoods capable of self-monitoring and information capture [21]. Data from all of these connected devices can be relayed to computers, tablets and smartphones across the room, or via the internet to colleagues at remote sites around the world.

Within this review, we will focus on how synthesis procedures can be assisted by visual information capture and processing. We will illustrate firstly how these methods can be used for simple laboratory and reaction monitoring, and more importantly, how these new technologies can be harnessed to inform and control further experimentation. In particular, we will describe iterative advancements towards the routine use of robotic and automation methods for safer and more sustainable machine-assisted processes [22–25]. We will not attempt to cover how digital camera methods in general can be applied to all chemical operations nor will we comment on other in-situ methods of reaction analysis such as UV, IR or Raman spectroscopy as this would constitute too broad a topic.

## Review

### From eyes to cameras

The combination of the human eye and brain sets the standard for recognising, recording and responding to the many stimuli in our daily lives. Our brains interpret this primary stimulus in extremely complex ways which we barely appreciate: for example, we have the ability to estimate the speed, direction and trajectory of a moving object with just a glancing look. This is in many ways a remarkable feat of pattern recognition and spatial cognition.

Within the laboratory environment, the art and craft of conventional synthesis involves many elements that rely heavily on visual stimuli. For example, reading a colour change when measuring a pH, identifying a phase boundary within a separation funnel, or assessing a component separation on a TLC plate. The innate pattern recognition skills that make these operations so trivial for us are a key distinguisher between a human and a computer, and so the development of alternatives to direct human observation has represented a significant challenge until now. As a result, these time- and labour-intensive operations, which are often the bottleneck in the research pipeline, continue to be carried out routinely by researchers and technicians.

Furthermore, traditional research facilities are extremely expensive to commission and run, and yet for a significant proportion of their lives they are under-used or even lying vacant. To overcome some of these inefficient practices, continuous processing methods such as flow chemistry [26–32] and other enabling technologies [16,33–35] are receiving increased attention.

With the continuing development of digital imaging technology, computer vision techniques and laboratory automation, some of these routine tasks may be delegated to a computer. Not only will this allow skilled researchers to spend their time more productively, but in cases where the changes involved are particularly fast, slow or otherwise difficult to perceive, the computerised “technician” may even be superior in some respects to its human operator. And indeed, in cases when our eyes are simply not capable of what is required, for instance, when we would like to observe the contents of a sealed reactor or events outside the visible range of the spectrum, we have no other choice but to rely on camera technology. The potential for computer processing means that digital cameras are more than just “eyes-on” equipment. The visual data produced are rich in information and can be used to perform complex calculations to make decisions and generate commands in real time.

**Figure 1:**
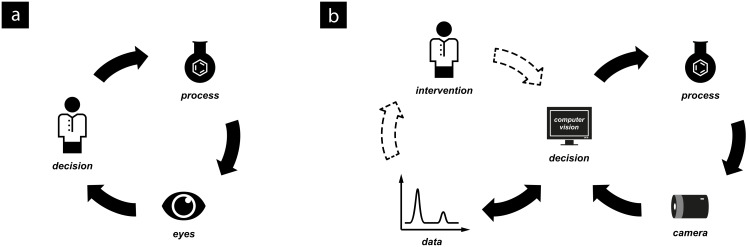
The evolution of computer-based monitoring and control within the laboratory of the future. (a) In the current status quo, successive iterations are reliant on human intervention. (b) In the laboratory of the future, the scientist is kept up to date at all times, and only need intervene manually if this becomes necessary or desired.

We consider the current state of laboratory decision making to require a human as an interface for allowing a reaction to progress, by performing a workup or a purification, or designing the next experiment (Figure 1a). By applying digital cameras and related technologies, this does not always need to be the case: We have identified a number of scenarios in which cameras have been employed by synthetic chemists to aid or enable their work. In the simplest cases, the camera provides the chemist with a view that would otherwise be inaccessible, or to make a recording of a long experiment to be played back later at a convenient time.

In more advanced cases, computer vision technology may be used to automatically process the images and provide data from an experiment in a numerical or graphical format for direct analysis. The implementation of algorithms to process the resulting data can allow some observations and thus decisions to be taken in an automated fashion (Figure 1b). As with any such technique, machine-assisted methods for data collection or synthesis procedures have a much reduced risk of human error, which may occur during long periods of classical observation or repetitive operations.

### Digital camera technology

**Figure 2:**
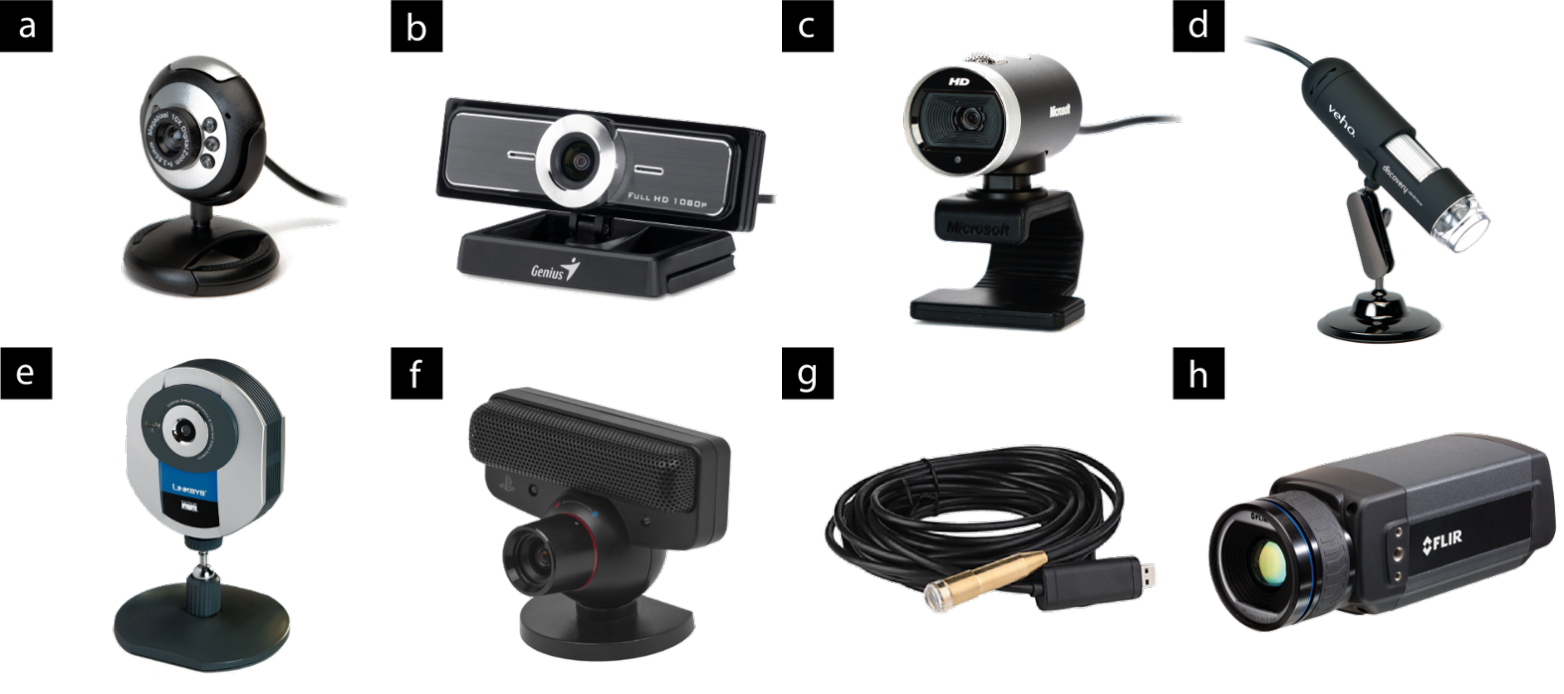
A selection of the wide range of digital camera devices available, focusing on those that can be attached directly to a computer for immediate streaming over a network. The capacity of current standard network connections imposes a practical limit on the resolution of imagery that can be transmitted to 640 × 480 pixels, i.e., 0.3 megapixels (MP). The increased resolution of high-definition (HD) imagery gives more information for computer vision purposes, but generally HD video needs to be resized (down-sampled) for real-time streaming. The prices stated are approximations of current listings. (a) Maplin Pluto Webcam: 0.3 MP USB camera with LED lights, £10; (b) Genius WideCam F100: 120° wide-angle USB HD camera, £40; (c) Microsoft LifeCam Cinema: USB HD camera, £40; (d) Veho VMS-001 Microscope: 1.3 MP USB camera with 20–200× magnification, £50; (e) Linksys IP camera: 0.3 MP wireless network camera, £100; (f) Sony PlayStation Eye Camera: high-speed (0.3 MP at 60 FPS; 0.1 MP at 120 FPS) 75° wide-angle USB camera, £25, requires free third-party software [36–37]; (g) Waterproof Borescope Home Camera: 0.3 MP USB camera with 5 m borescope and LED illumination, £20 [38]; (h) FLIR A305sc: 0.3 MP thermal imaging network camera, £6,800.

There is a vast selection of digital cameras available commercially, including smart phones, webcams, SLR cameras, and high-speed or high-resolution devices, with a correspondingly wide range of prices (Figure 2). It is therefore wise to take great care in selecting the unit that is most appropriate for the desired application. As we will explain, such applications can range from the capture of static images or time-lapse sequences, to real-time video monitoring, to the capture of ultra-fast processes. Digital cameras are highly suited to the role of recording laboratory events, because the images and videos produced can be streamed to computers, tablets and smartphones in real time, and simultaneously stored for later retrieval and processing.

The solid-state nature of CCD (charge-coupled device) and CMOS (complementary metal–oxide–semiconductor) imaging chips means that digital cameras can be very robust, which is of significant benefit in a laboratory environment. Many devices are highly portable, being hand-held or even smaller in size, allowing rapid reconfiguration and minimising the impact on an experimental setup. Specialist devices can operate beyond the normal visible regions, providing for instance near-IR imagery for low-light situations or IR thermal imagery for monitoring exothermic events and microwave chemistries [39–41]. Such equipment usually comes at a significantly increased cost. The variety of available units speaks for the range of potential applications, such as those we will describe in this review.

### Laboratory monitoring

General laboratory monitoring can help to provide a safer working environment or enable effective coordination of multiple experiments with minimum effort. With this in mind, a system of remotely accessible cameras is an important addition to any laboratory. With issues of work versus lifestyle management becoming increasingly important, and to obtain the most efficient use of space and the opportunity for a 24/7 working regime, the ability of digital cameras to provide real-time information on the state of a laboratory is particularly useful.

**Figure 3:**
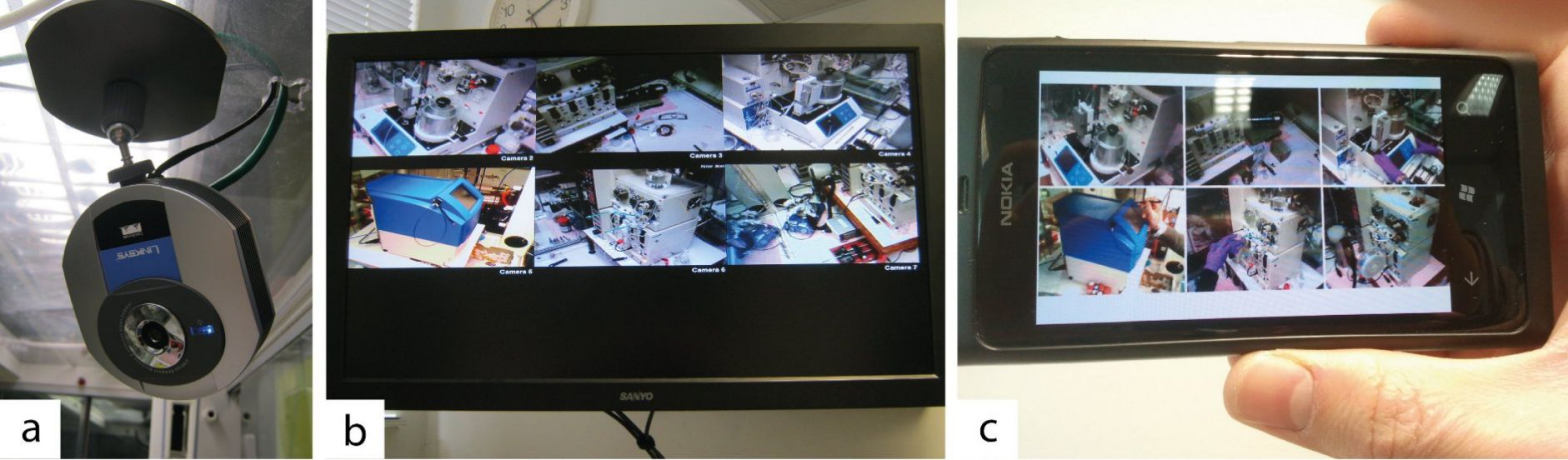
(a) Network cameras (Linksys WVC54GC) in operation in the Innovative Technology Centre laboratory. (b) Images can be displayed on a television in a connected office, or (b) accessed remotely through mobile devices.

When we established our Innovative Technology Centre (ITC) for advanced chemical synthesis in 2005, all of the fume-hoods and equipment were monitored by networked cameras mounted such that they captured a view of operations underway in a busy laboratory (Figure 3). The video footage was displayed on a split-screen TV monitor for all to see in the research offices, and could be accessed remotely on mobile telephones connected to the internet.

**Figure 4:**
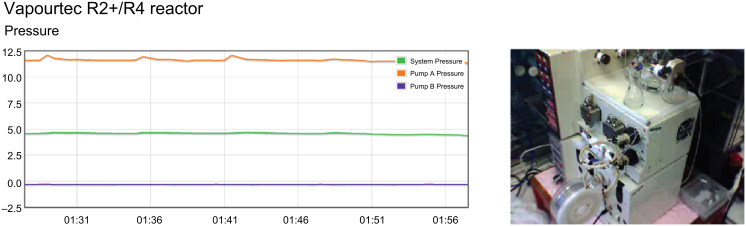
Remote transmission of video imagery and reaction monitoring data.

Although the original purpose of these cameras was for general observation to improve safety, they can also be remounted to monitor individual apparatus for more specific tasks; for example, those described in later sections of this review. Additionally, visible information of the state of laboratory apparatus can be displayed alongside data feeds to provide an additional layer of remote observation (Figure 4) [18].

### Camera-assisted vision

**Figure 5:**
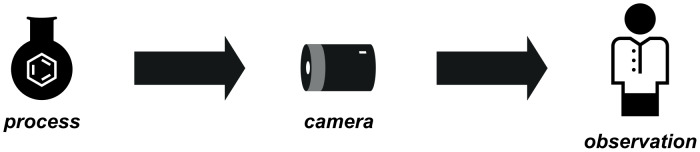
A camera can assist the chemist in a number of ways. Digital video recordings of reactions can be useful for recording a procedure, for capturing images at high speed or outside the visible regions of the spectrum. In more advanced cases, a digital camera can record a long reaction for later review, or provide visual access to a reactor setup that might not otherwise be available.

Many scientific endeavours lend themselves well to photographic recording (Figure 5). The digital camera serves us particularly well during chemistry experimentation where one may wish to record colour changes, crystallisation, precipitation, viscosity and other phase changes, or detect the onset of polymerisation and gas evolution. Indeed, there are many examples in the literature whereby this basic level of monitoring and recording can be beneficial.

**Figure 6:**
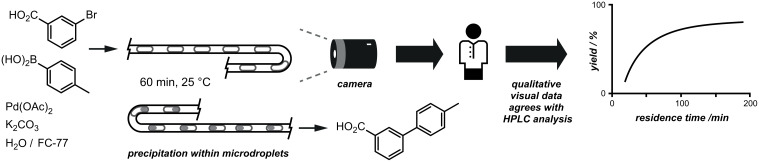
Suzuki–Miyaura reaction performed within a microfluidic system. The product is observed by high-speed microscope photography, which shows a precipitate forming within the microdroplets.

For example, monitoring of chemical events within microdroplets in flow has resulted in many emerging techniques for sophisticated analysis, timing and control [42–44]. High-speed cameras provide excellent visual information for evaluating such reactions. Huck and co-workers recently reported the application of the synthetically powerful Suzuki–Miyaura reaction within aqueous microdroplets buffered by catalytically active fluorous interfaces [45]. Images of the flow channels captured by a high-speed camera provided preliminary kinetic data by allowing the precipitation of the solid product within the microdroplets to be visualised accurately (Figure 6).

**Figure 7:**
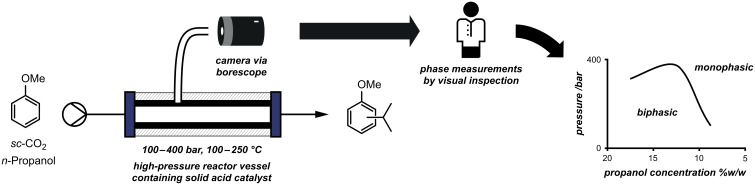
Friedel–Crafts reactions performed by using solid-acid catalysis at high pressures. A camera allowed the interior of the reactor to be visualised so that phase measurements could be taken.

Another beneficial way to use a camera to assist in a reaction optimisation is simply to see what is happening in an otherwise inaccessible reaction vessel or closed cavity. During studies on the Friedel–Crafts alkylation of anisole with various solid-phase Brønsted acid catalysts in supercritical carbon dioxide (Figure 7), Poliakoff and co-workers studied the effect of varying the concentration of the organic reagent in the liquid CO_2_ solvent [46]. The high pressures required (100–400 bar) stipulated the use of a sealed reaction vessel, and consequently they employed a borescope camera to visualise the interior of the reactor. This allowed the authors to make phase measurements by visual inspection, to discover that with *n*-propanol as the alkylating agent, the best results for monosubstitution were obtained by using either Amberlyst 15 or Purolite CT-175 at temperatures between 100 and 150 °C.

In another very nice example of the use of cameras to assist with organic synthesis, Leadbeater and co-workers report an application in microwave chemistry [47]. As all synthetic chemists are aware, laboratory microwave equipment provides a shielded reaction chamber for conducting preparative chemistries at high temperatures and high pressures usually in batch mode in sealed vessels or in some cases in flow [40,48]. While it is easy to record reaction times, temperatures and pressures by using the inbuilt sensors, this is a perfect example of how the very nature of the equipment can preclude direct observation of the reaction cell. Events such as precipitation, colour changes, and even vessel stirring, which can be invaluable metrics for the extent of the reaction, are seemingly beyond reach.

**Figure 8:**
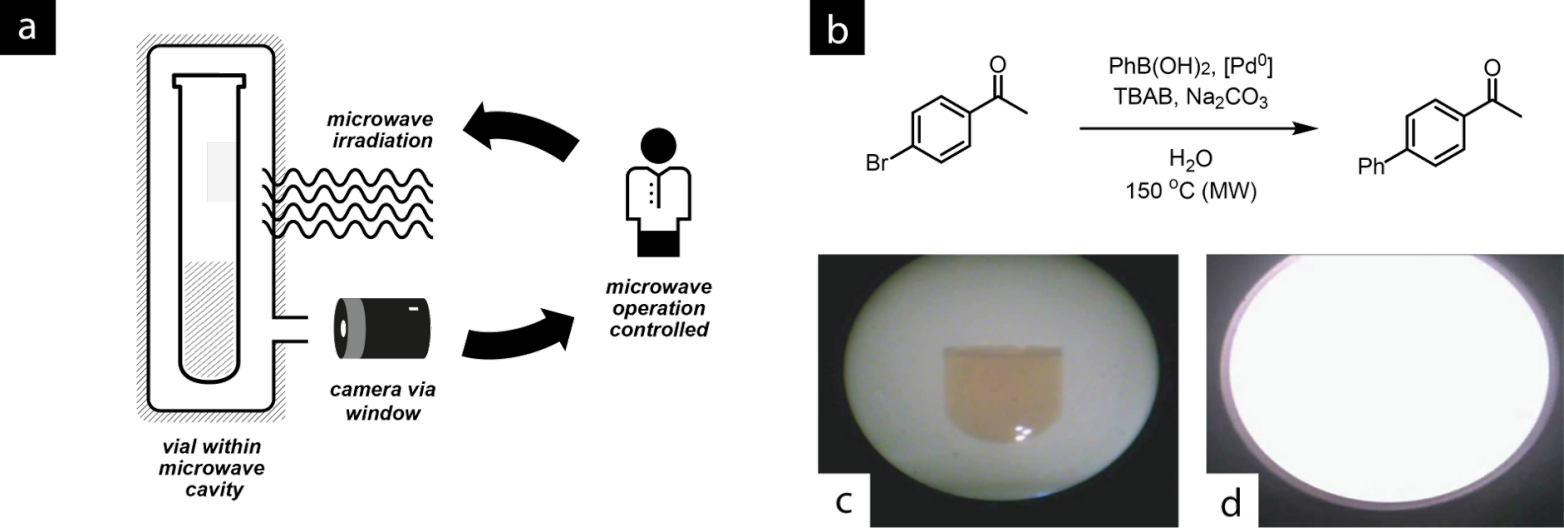
(a) The video camera setup providing a view of the reaction within the microwave cavity; (b) a palladium-catalysed Suzuki coupling performed by using this arrangement; (c) the reaction vessel is visible with the aid of the video camera; (d) a still from the footage showing arcing observed as a bright flash of light. Video stills reprinted from [47], Copyright 2008, with permission from Elsevier.

However, by illuminating the reaction cavity with a white light LED and placing a digital camera with its lens outside the cavity walls but with access to the reacting chamber through a small port, the reaction profile can be observed by digital video (Figure 8). The 1.3 mexapixel CCD produced good images of the reaction vessel, so that several standard microwave reactions could be monitored with this set-up. Video images are available with the supplementary information of that publication [47].

Importantly, the camera could also be used to improve the safety of microwave processes. The authors note that when the stirring function of the microwave was disabled during a metal-catalysed cross-coupling reaction, aggregated palladium metal deposited on the glass surface of the vial and led to arcing within the microwave cavity; an effect that was quite obvious on the video footage (Figure 8d). The authors point out that such metal deposition can also result in localised melting of the glass and the potential for pinhole fracture of the reaction tubes. The camera therefore permits detection of impending failure and allows the experiment to be terminated prior to a catastrophic event. The use of a camera-augmented microwave reactor has also been reported by Kappe and co-workers for the observation of arcing during the irradiation of a number of metal-solvent systems [49], such as during the formation of Grignard reagents [50], and to monitor stirring effects within the microwave chamber [51].

**Figure 9:**
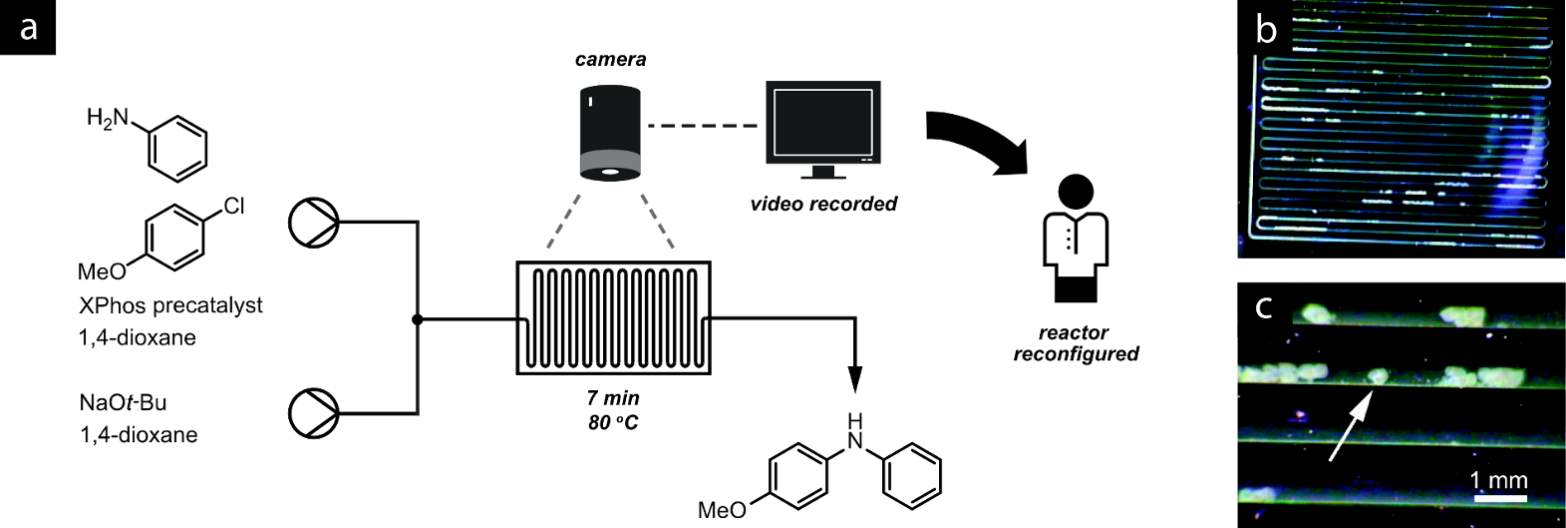
(a) Buchwald–Hartwig coupling within a microchannel reactor. (b) Camera view of aggregate deposits forming in the glass chip. (c) An expanded view of the camera image. Images reprinted with permission from [52]. Copyright 2010 American Chemical Society.

High-speed digital camera footage was used by Jensen and co-workers to investigate metal aggregation in a palladium-catalysed cross-coupling reaction [52], this time within a microreactor where such aggregation can lead to pressure spikes and reactor plugging (Figure 9). Data collected using this monitoring technique allowed them to investigate the methods of aggregate formation. Using this information, they employed acoustic irradiation to eliminate channel bridging, and flow rate control to manage channel constriction.

Others have also recognised the benefits of ultrasound techniques, for example, during palladium catalysed amination reactions [53–54], photodimerization studies [55], MnO_2_ oxidations [56] and during phase-transfer reactions [57]. The use of high-resolution cameras to specifically examine physical effects during the merging of sonochemistry and microfluidic techniques, leading to improved reactor design, was the subject of a recent feature article [58] reviewing developments in this rapidly expanding area.

**Figure 10:**
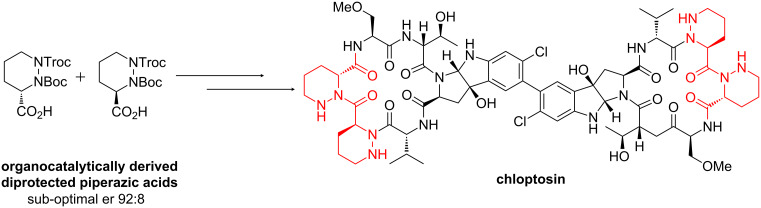
The key diprotected piperazic acid precursor in the synthesis of chloptosin.

In our own research group, a camera recording was extremely useful during the preliminary stages of the total synthesis of a dimeric cyclic hexapeptide with anti-tumour properties, chloptosin [59–60]. The route required a significant quantity of an orthogonally protected piperazic acid (Figure 10). This was achieved using a new enantioselective organocatalytic protocol with a tetrazole organocatalyst, which afforded dihydro pyridazines from achiral aldehydes [61–62]. Unfortunately, while this procedure did provide access to material on a suitable scale, the enantiomeric ratio between the final diprotected piperazic acid isomers was deemed to be unsatisfactory for the remainder of the total synthesis, and enantiomeric upgrading was required.

**Figure 11:**
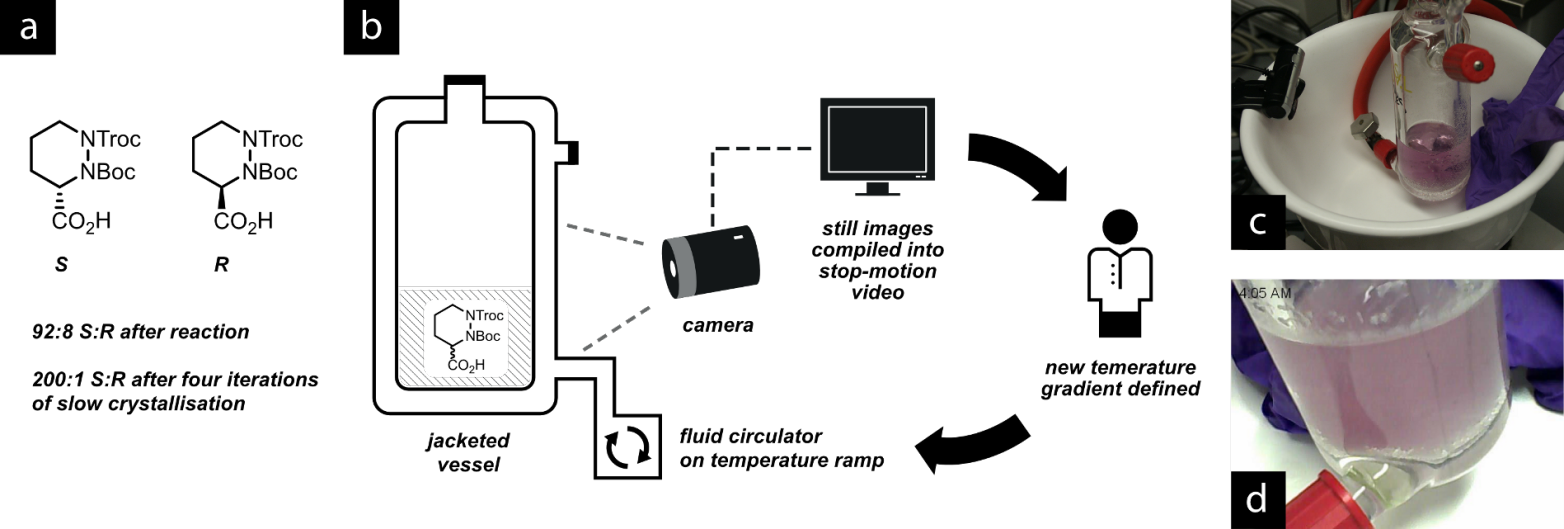
(a) Piperazic acid mixture, and (b) apparatus for enantiomeric upgrading by recorded crystallisation. The thermostat slowly adjusts the temperature of the jacketed flask containing the piperazic acid over several hours. (c) A digital camera records images of the flask as it crystallises over 12 hours. A dark background allows the precipitation to be observed. (d) An example of the images captured by the camera. These are stored on the computer for later assembly into a video sequence (see Supporting Information File 1).

Standard recrystallisation techniques proved to be unsuccessful for the upgrading process, and therefore we opted to perform a slow crystallization process over 12 hours, which was recorded by a digital camera taking still images every 60 seconds (Figure 11). The images were combined into a video (for an example, see Supporting Information File 1), which was played back to identify the temperature at which crystallization began. This information was used to iteratively improve the temperature gradient to obtain slower and slower crystallisation. This process eventually afforded material with an enantiomeric ratio in excess of 200:1, which was suitable for the remainder of the synthesis.

In a later project, we used a digital camera to record the operation of a prototype magnetic-field-induced flow mixer device for in-line continuous-stream processing [63]. Reviewing the video recording at the end of a long run allowed us to confirm that the degree of mixing was constant for the required time. Supporting Information File 2 shows the device in operation.

**Figure 12:**
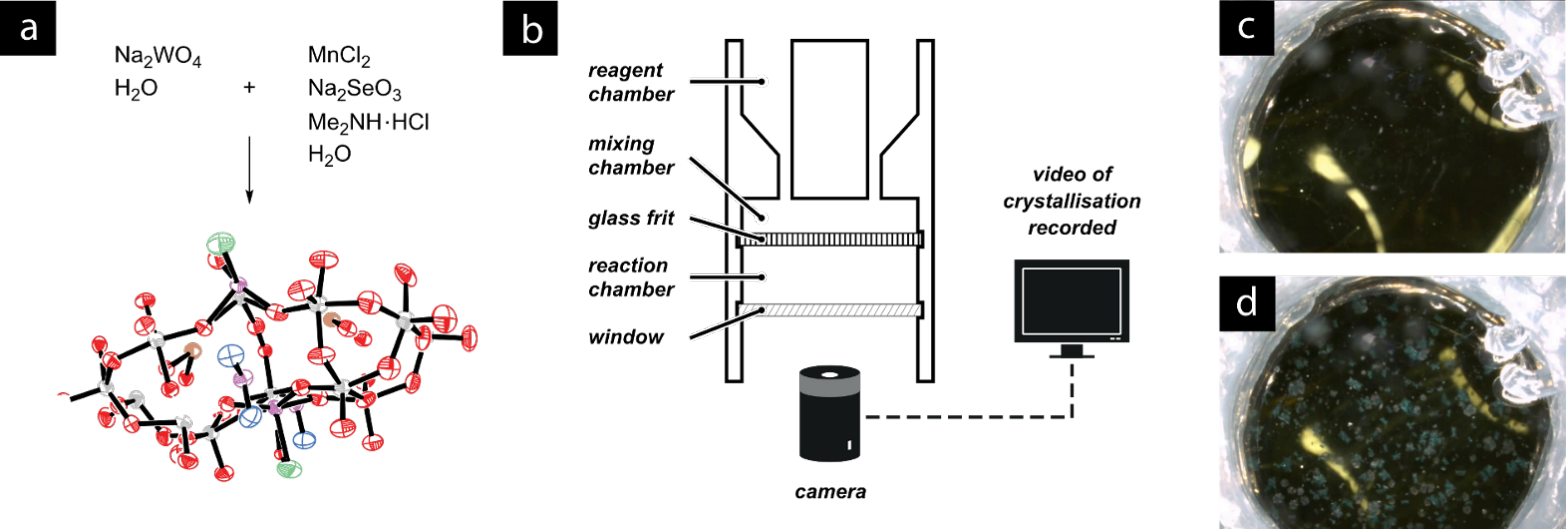
(a) Crystallisation of a Mn(II) polyoxometalate. (b) A bespoke reactor produced using additive fabrication. A window integrated into the reactor enables a camera to record a video of the crystal formation process. (c) Still images before and (d) during crystallisation. Still images reprinted with permission from Macmillan Publishers Ltd: Nature Chemistry [64], copyright 2012.

Cronin and co-workers have recently reported the use of three-dimensional design software and an open-hardware 3D printing device to produce low-cost bespoke reactionware for applications in both organic and inorganic synthesis [64]. For some metal-complex formation procedures, the digital blueprint of the custom reactor was modified to incorporate a transparent port, through which the crystallisation could be recorded using a digital camera (Figure 12). This allowed the size of the crystals and the rate of crystallisation to be observed through an otherwise opaque reactor.

### Computer vision

**Figure 13:**
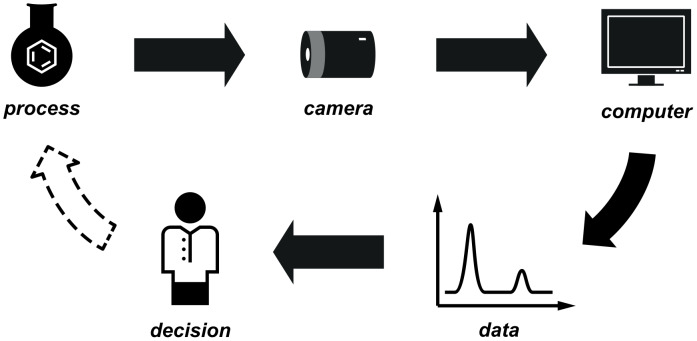
Computer processing of digital imagery produces numerical data for later processing.

As well as simply seeing the traditionally inaccessible or speeding up the laborious process of watching a slow crystallization, this concept can be taken one step further by programming a computer to process the recorded images automatically to produce data. As with any computerised process, not only does this free up the researcher but the data collected is inherently less variable, since there is no longer a human factor in the experimental error. The data can then be interpreted and fed back to the system in a relevant way (Figure 13).

**Figure 14:**
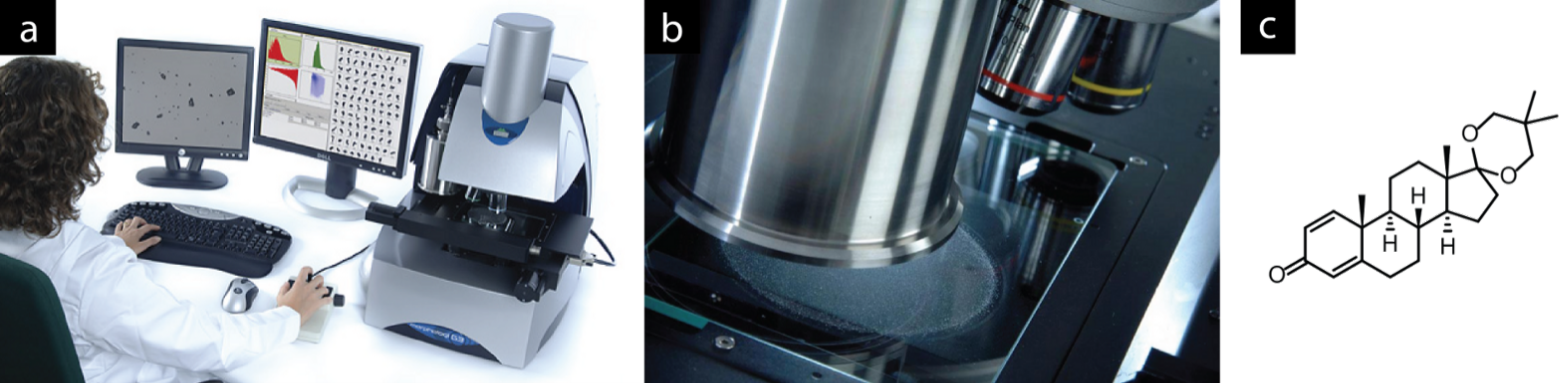
(a) The Morphologi G3 particle image analyser, which uses images captured with a camera microscope (b) to measure particle size, shape and count; (c) the pharmaceutical intermediate androsta-1,4-diene-3,17-dione, cyclic 17-(2,2-dimethyltrimethylene acetal) whose crystallisation was studied. Photographs reprinted with permission from Malvern Instruments, Inc.

In some cases, highly specialised software is applied for the characterisation of experimental imagery. On an industrial scale, reproducible crystallisation is a crucial method for purification, and many physical parameters for the crystallisation process can be captured. The shape distribution of nascent crystals is one such parameter that is most effectively measured visually. For example, a particle characterisation system and associated software developed by Malvern Instruments, Inc. [65–66] is routinely applied to analyse particle sizes (Figure 14a,b).

One area in which enabling technologies make a significant impact is when a process is taken from the laboratory to an industrial scale. In a recent publication by Kadam and co-workers [67] a number of process analytical technologies (PAT), including visual monitoring of the crystal size and shape distribution calculated by using Malvern software, were used to optimise the crystallisation of an advanced pharmaceutical intermediate (Figure 14c) directly on an industrial scale. This eliminated the need for time-intensive process development at intermediate scales; the authors state that the development of purification processes in parallel with modular control strategies such as particle analysis leads to a consistent product quality in the final procedure.

Until recently, the development of complex bespoke software solutions would not normally be considered a feasible option, particularly in the academic environment. However, the availability of powerful open-source image processing software such as the Python Imaging Library [68] (PIL, an image processing library for the Python [69] programming language) and OpenCV [70] (a C++ library for creating real-time computer vision applications with bindings for the Python language [71]) enables powerful image-recognition logic to be harnessed for a low cost and by nonspecialists.

Access to these libraries is only one contributing factor: such software projects are often well documented with a number of examples, and the internet provides a medium for rapidly sharing and recycling code and applications. Another important aspect of software libraries such as these is that whilst they use highly efficient C or C++ code to perform processor-intensive calculations, the “bindings” allow their full functionality to be harnessed by using languages such as Python, which have been designed to enable rapid development. Additionally, the library often takes care of “low-level” functions, such as connecting to a camera across a USB connection. This makes access to the powerful analytical methods as easy as possible.

One of the more straightforward methods to interpret an image programmatically is to monitor colour changes. By the very nature of digital-image file formats, the colour of each point (pixel) of an image is encoded numerically [72] according to a particular colour model. One such colour model is RGB, in which each pixel is broken into red, green and blue components. Based on the anatomy of the human eye, this system was designed for electronic systems such as computer displays, which often consist of a matrix of tiny red, green and blue points, whose brightnesses can be controlled individually to make up the overall picture.

Sometimes it is more convenient to use a different representation of the colour space, such as HSV (sometimes referred to as HSB for hue, saturation and brightness), which is a cylindrical-coordinate representation wherein the hue of a colour is encoded as an angle around the colour wheel. This allows regions of a particular hue to be extracted from an image. The strengths and limitations of individual colour models and their representations is itself a complex science; fortunately, such knowledge is generally not required for computer vision applications.

**Figure 15:**
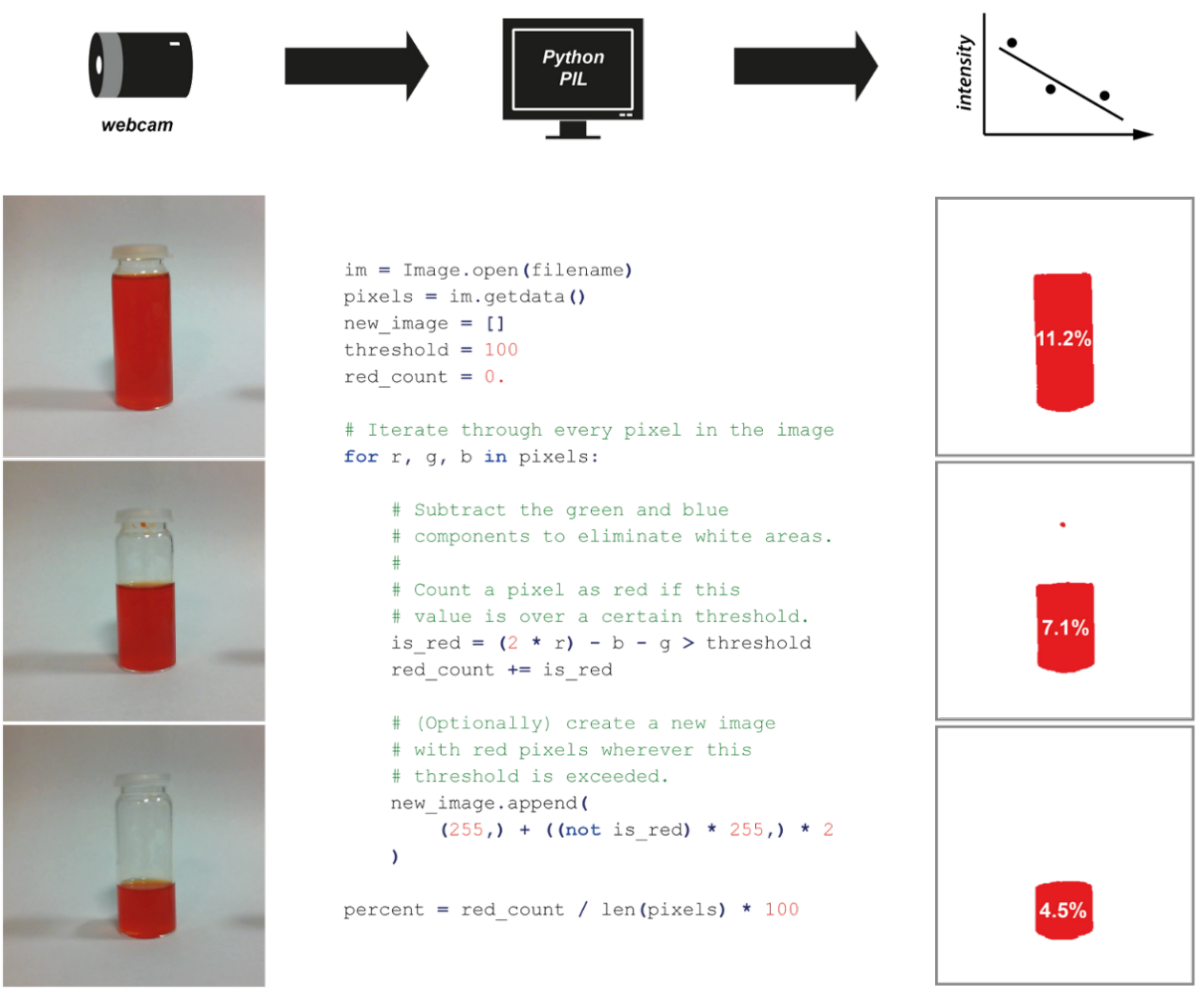
Use of the Python Imaging Library to analyse the proportion of an image consisting of red pixels. An image is read into the program from a saved file (reading from a USB or networked camera is also possible) and separated into its constituent pixels, whose component colours are then analysed to identify those that are predominantly red in colour. The percentage area of the image that is red is then calculated as the proportion of pixels that surpass a threshold. In an 8-bit image each colour component is encoded as an integer from 0 to 255, and so the threshold of 100 here corresponds to approximately 40% red character. This is sufficient to pick out the region corresponding to the red liquid in the images.

Figure 15 illustrates a trivial example of colour identification, using the basic RGB colour space. Three images of a sample vial are analysed for the number of red pixels present. A red pixel has a red component significantly higher than its green or blue components, whereas white or grey pixels have approximately equal red, green and blue components. Therefore, subtracting the blue and green components from the red ones filters out the white regions. In this case, the percentage area of the red region correlates with the volume of liquid in the vial. In a real situation, a numerical output such as this percentage can be recorded for later analysis or used immediately for automated decision making.

**Figure 16:**
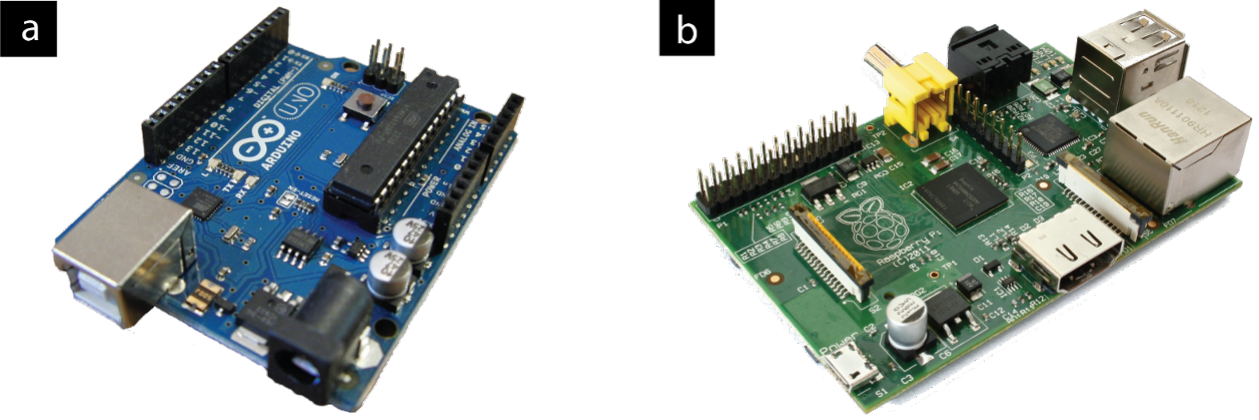
(a) Arduino [73,75], a flexible open-source platform for rapidly prototyping electronic applications. (b) Raspberry Pi [74,76], a low-cost Linux-based computer system with a small size and power footprint ideal for embedded applications. Both devices retail for under £30.

Such technologies are sufficiently practical and economical to permit widespread use, not just confined to academic laboratories. With the ready availability of cheap CCD devices and computational platforms such as Arduino [73] and Raspberry Pi [74] (Figure 16), the image capture and computer processing can be integrated into commercial devices for a fraction of the cost of an equivalent visible-light spectrometer. The developing shift from power-hungry desktop processors towards low-power, portable, yet still powerful, processors designed for smartphones and tablet computers (such as the ARM-based processor found on the Raspberry Pi) allows computational platforms to be more mobile in nature and thus better suited for deployment within the synthesis laboratory environment.

**Figure 17:**
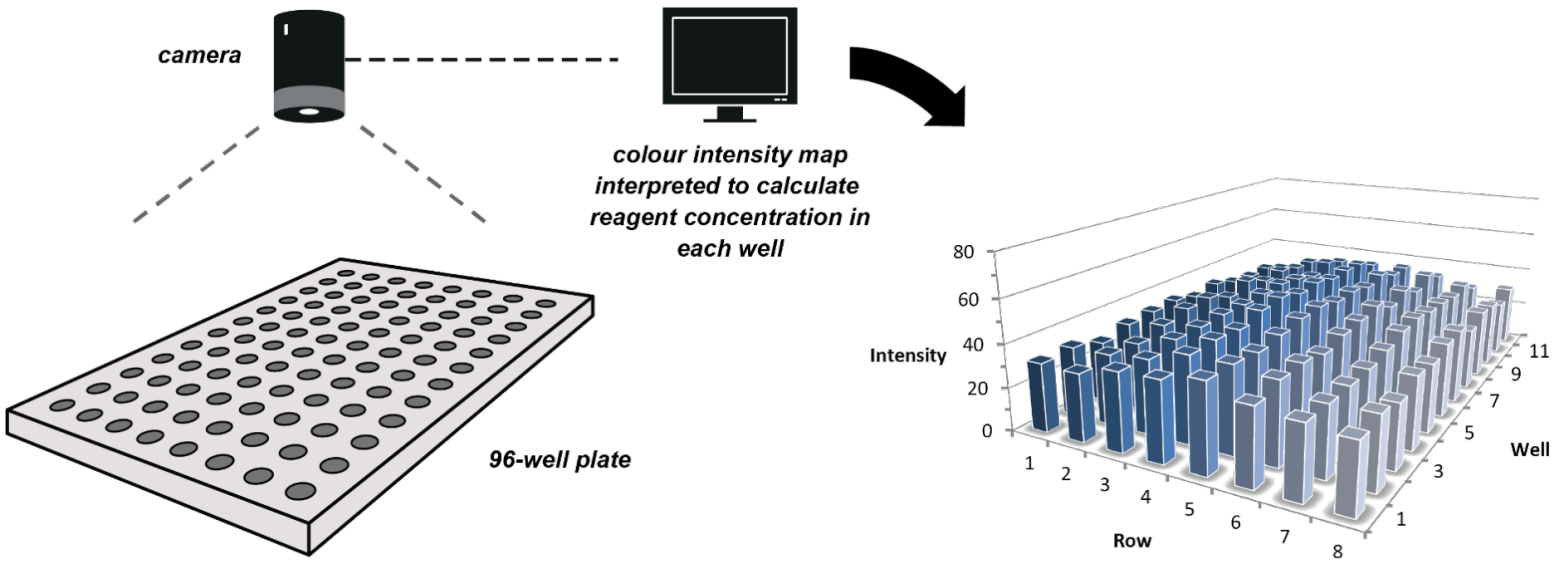
Patented device incorporating a standard 96-well plate illuminated by a white-light source. The plate is observed by a CCD camera, such that multiple reactions involving a colour change can be monitored simultaneously. The use of a solid-state CCD imager reduces the number of moving parts, which should improve the overall reliability of the device.

In a recent patent application [77] the authors disclose a device for monitoring chemical reaction mixtures. Reactions are performed in a standard 96-well plate illuminated by an LED light and imaged with a CCD camera. In an example reaction, the intensity of an orange colour indicating the release of a dimethoxytrityl blocking group was measured, allowing parallel monitoring of a plurality of chemical reactions in an array format (Figure 17). This system is specifically targeted at oligonucleotide synthesis where multiple reactions may frequently be performed in parallel, and so this invention included the development of optical analysis software to process the vast amount of data collected. Observing visible colour properties (instead of UV absorbance, for example) in this way allows a cheap CCD camera to be used in place of an expensive spectrophotometer.

Our own group has recently developed a reactor for the introduction of gases into liquid streams in a controlled manner. The early stages of development were heavily influenced by a consideration of the potential for automated visual monitoring, and indeed this visual monitoring technique co-evolved with the gas-flow reactor as we assessed different gases and their potential reactivity in continuous flow processes. The reactor consisted of a section of semipermeable polymer tubing suspended in a reagent bottle containing an atmosphere of reactive gas (Supporting Information File 3). When a liquid stream was passed through the tubing, the microporous nature of the tubing used (Teflon AF-2400) allowed gas-to-liquid transfer [78].

**Figure 18:**
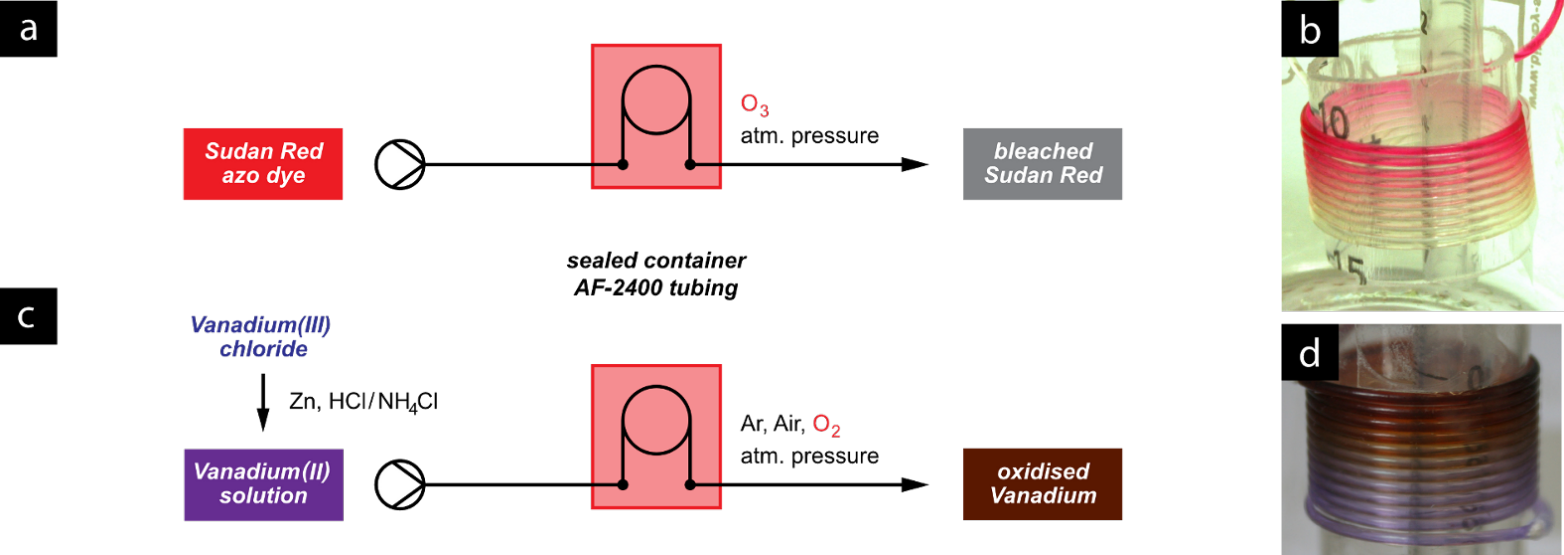
Simple colour-change experiments to assess a new AF-2400 gas permeable flow reactor. The reactor consists of a sealed bottle (a) Sudan red dye is bleached by ozone gas permeating into solution through the reactor tubing, which visible (b) as a colour change from red to colourless. Similarly, solvation of oxygen gas (c) is followed by the oxidation of vanadium(III), which also has a distinct colour change from purple to brown (d).

Initially we used the phenomenon of a simple colour-change reaction to observe the permeation of ozone gas through the AF-2400 tubing. By injecting solutions of the azo-dye Sudan Red into a section of this tubing housed within a jar that was purged with ozone, the red colour was visibly bleached over a period of time (Figure 18a). This meant that the dissolution of ozone gas through the membrane could be followed directly through the walls of the glass reactor by observing the decolourisation of the dye. This led to an initial assessment of good parameters for the design of the reactor, such as the length of tubing used and the residence time required to effect a complete reaction.

For subsequent work using oxygen gas, we moved to a more elegant but still simple tube-in-tube reactor setup [79]; in this case the oxidation of a lilac solution of vanadium(II) was used to indicate the presence of oxygen in the solvent stream. With increased concentrations of oxygen in the reactor atmosphere, this oxidation proceeded with faster rates (Figure 18b).

**Figure 19:**
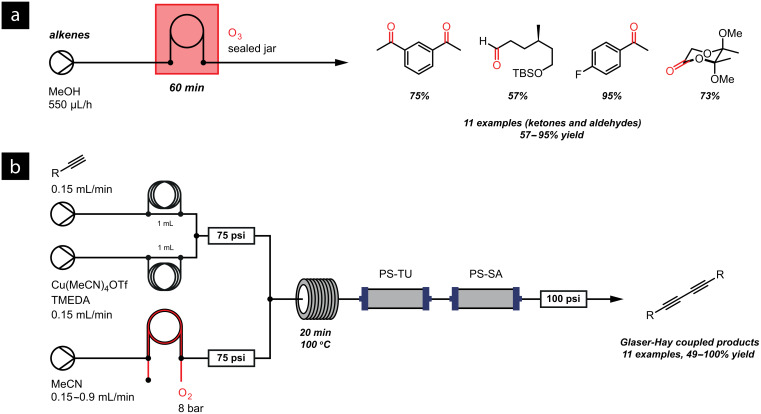
(a) Ozonolysis of a series of alkenes using ozone in a bottle-reactor; (b) Glaser–Hay coupling using oxygen gas in the second-generation reactor. Cartridges containing polymer supported (PS) reagents are used to scavenge residual reagents: thiourea (TU) sequesters copper, and sulfonic acid (SA) captures the TMEDA base.

Having determined that the gaseous reagents were passing through the semipermeable tubing, we were able to use the tube-in-tube reactor for preparative synthetic chemistry. Ozone was used to oxidatively cleave a variety of alkenes whereas oxygen was used to aid the oxidative homo-coupling of terminal alkynes (Figure 19).

Our use of camera imagery became more sophisticated as we began to acquire data on the precise concentrations of the gases in the tube-in-tube reactor. In a recent publication describing the use of ammonia gas in flow synthesis [80], a reversed, “tube-in-tube” reactor configuration was employed whereby the gas was introduced into the semipermeable tubing, while the substrate passed through a second, outer, PTFE tube. This arrangement facilitated better control of temperature within the system, which had been found to be necessary for reactions using ammonia. Furthermore, by confining the gas within a tube, the volume present within the reactor at any time is significantly reduced, which is an important consideration if more hazardous gases are to be used.

**Figure 20:**
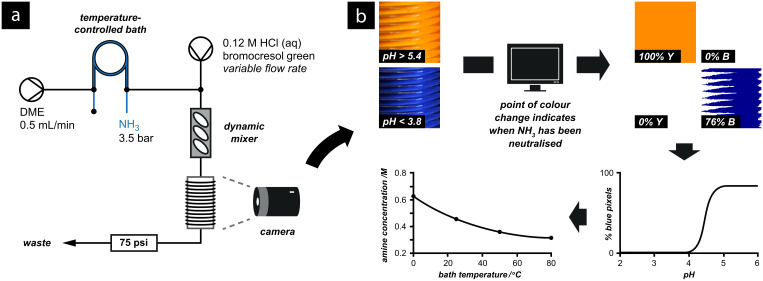
(a) Camera-assisted titration of ammonia using bromocresol green. NH_3_ is dissolved in the gas-flow reactor inside a temperature-controlled bath, and then mixed with varying proportions of acid. (b) The point of neutralisation is observed by a colour change from yellow to blue: The camera collects images which are analysed by a computer. The image is split into its “blue” and “yellow” components and the relative proportions compared. (A pixel is considered to be blue if (2*r* – 2*b* > 140); and yellow if (2*b* – 2*r* > 140) where *b* and *r* are the blue and red values of the pixel, respectively). The concentration of NH_3_ dissolved within the reactor at each bath temperature can then be calculated from the pH at which the stream is neutralised.

In this case, the reactor configuration was calibrated by a colourimetric calibration using another dye, bromocresol green (Supporting Information File 3 shows an acidic solution of bromocresol green changing colour under an ammonia atmosphere). The ammonia-enriched solvent stream was continuously mixed with a solution containing the indicator and hydrochloric acid, passing through a dynamic mixer to ensure good homogeneity of the resulting solution. The combined streams were directed into an observation coil, in which the colour of the indicator could be captured using a digital camera (Figure 20). Varying the flow rate of the acid stream altered the pH of the combined outflow.

The bromocresol green indicator underwent a distinctive change from orange to blue when the concentration of ammonia exceeded the concentration of added acid. By monitoring for a switch in the colour from blue to yellow as the flow rate of the acidic stream was varied, the concentration of dissolved ammonia at a particular temperature of the heating bath could be measured to a good precision. We envisage that with precise computer control of the pumps and real-time interpretation of the camera images, more accurate titrations and other reaction controls could be implemented.

Another computer-assisted method was employed when working with hydrogen gas. Hydrogen has a low solubility in many solvents making accurate gas dissolution measurements difficult and potentially leading to dangerous out-gassing. Hydrogen reactors such as this one have proven to be very useful for reduction reactions [81–82], with homogeneous, heterogeneous and asymmetric [83] hydrogenation possible.

Conventional methods of gas solubility measurement often involve measurement of pressure differences on dissolution. Using typical apparatus for such measurement, equilibration times can often be very long, i.e., sometimes several hours, due to the relatively low surface-to-volume ratios involved [84]. To demonstrate that gas permeation and dissolution into the reacting flow stream followed Henry’s law and that rapid saturation was achieved, a computer controlled “bubble-counting” technique [85] was employed. This method is analogous to the use of a burette, but offers an advantage over traditional methods in that information can be relayed in real time as adjustments are made to the experimental parameters.

**Figure 21:**
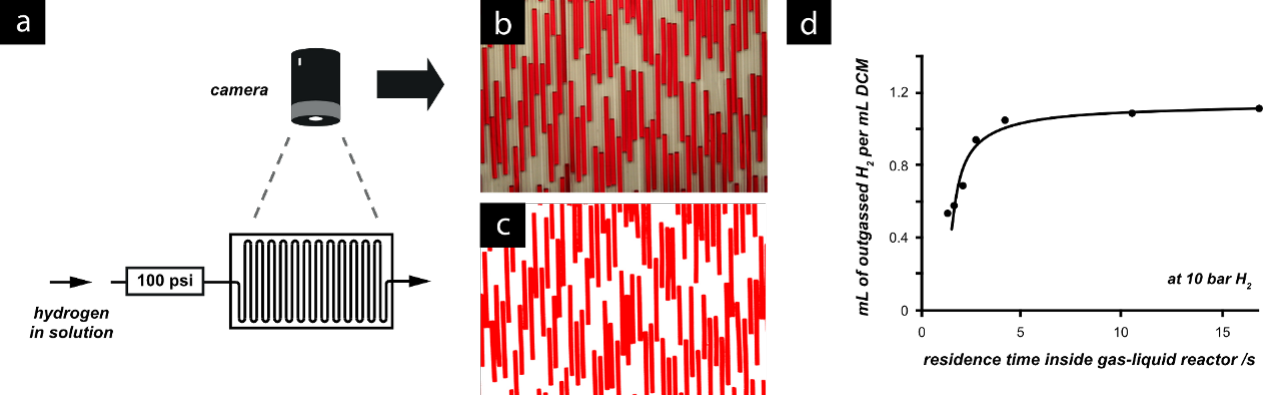
(a) Bubble-counting setup. As the output of the gas-flow reactor (hydrogen dissolved in dichloromethane) passes into a low-pressure tubing array the hydrogen gas comes out of solution, forming bubbles; (b) the camera records images of the biphasic flow; (c) the images are processed to identify and count the red pixels; (d) graph showing the quantity of hydrogen in solution. In this case the amount of hydrogen is found to saturate after 5 seconds within the gas–liquid reactor.

After passing through the gas-flow reactor a flow stream of dichloromethane containing a red dye was allowed to degas in a lower pressure environment. A camera mounted over a flat tubing array captured images of the resulting biphasic system. The images were automatically filtered to locate the areas of coloured solvent. By counting the relative proportion of red to white regions, the amount of gas in the solution could be quantified (Figure 21).

The approach was then used to demonstrate that the same semipermeable membrane could be used in a second device to efficiently remove excess unreacted hydrogen, enabling further reactions or downstream processing.

Incorporating the processing capabilities of computer vision software with digital cameras represents a large step towards the goal of automating routine synthetic tasks. With continued development and exposure these techniques will be more commonplace in the laboratories of tomorrow.

### Real-time video broadcasting enabling remote reaction control

We have discussed how digital cameras and recordings can give the synthetic chemist an otherwise accessible view of a reaction or process. Sometimes visual access is limited by distance alone: for example, traditionally a chemist must relinquish control over a reaction to a colleague or to fate when he or she leaves the laboratory in the evening, or even just momentarily throughout the day.

**Figure 22:**
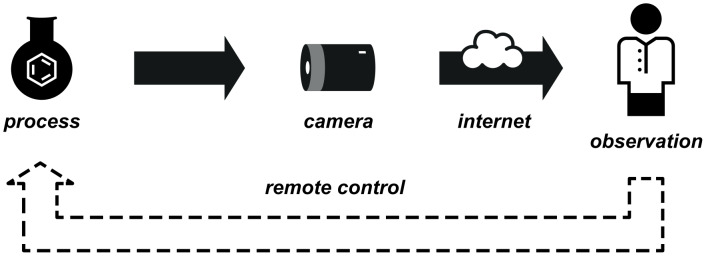
Usage of digital cameras to enable remote control of reactions.

The proliferation of wireless internet access and the ready availability of technology for transmitting images and video in real time, combined with reactor apparatus with the potential for remote control means that this limitation can be overcome (Figure 22).

During a challenging programme towards the synthesis of imatinib [86–87], some operations benefited substantially from remote monitoring by digital video camera.

**Figure 23:**
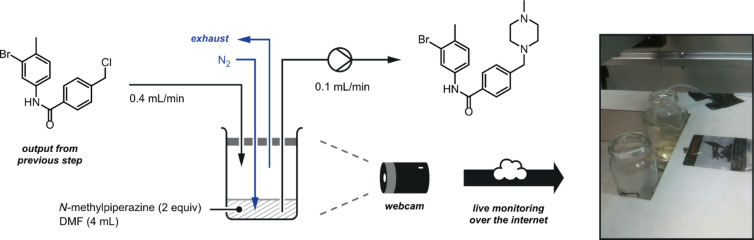
In-line solvent switching apparatus. The reactor output is directed into a bottle positioned on a hotplate. A flow of nitrogen gas removed the heated DCM solvent leaving the desired intermediate in DMF (a less volatile solvent). A webcam is directed at the evaporation setup so that it can be monitored remotely.

An inline evaporation apparatus was developed to perform a solvent switch from dichloromethane (DCM) to dimethylformamide (DMF). The reactor output was dripped into a heated vial containing DMF where a flow of nitrogen gas removed the DCM solvent (Figure 23). A pump removed the concentrated solution through another tube. This unit could be constantly visualised by using a webcam to ensure that no overfilling of the vessel occurred.

**Figure 24:**
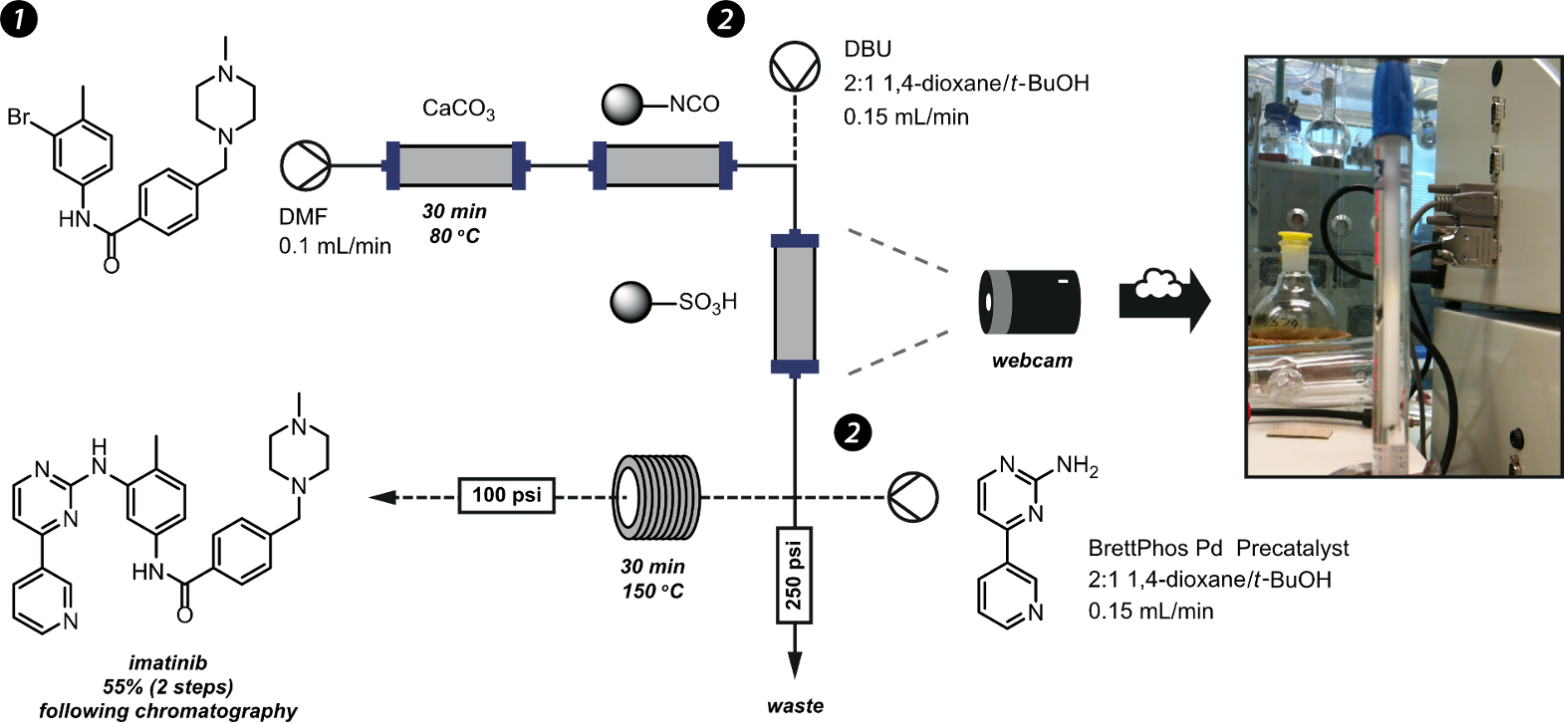
Catch and Release apparatus. (1) The amide intermediate is sequestered onto the central sulfonic acid column. A colour change indicates the extent of this process; by accessing pictures from the webcam remotely the reactor can be shut off at the end of the reaction in order to save energy and solvents. In the next step (2) the amide is released by washing the column with a base (DBU) and directly used for a cross-coupling to complete the synthesis.

In the following step, the coupling of a benzylic chloride with *N*-methylpiperidine gave a product, which was sequestered onto a sulfonic acid (QP-SA) scavenger cartridge. After washing away any impurities, pure material was then released with a base in order to perform the final palladium-catalysed coupling process (Figure 24).

Passage of the reacting solution through two columns of immobilised reagent caused significant dispersion of the product, resulting in a long and unpredictable time required to fully sequester the intermediate. Fortunately, as the product was captured onto the sulfonic acid, the appearance of the functionalised silica support changed from partially translucent to opaque. This gave a good visual illustration of the extent to which the product had been trapped, and hence the progress of the flow stream.

**Figure 25:**
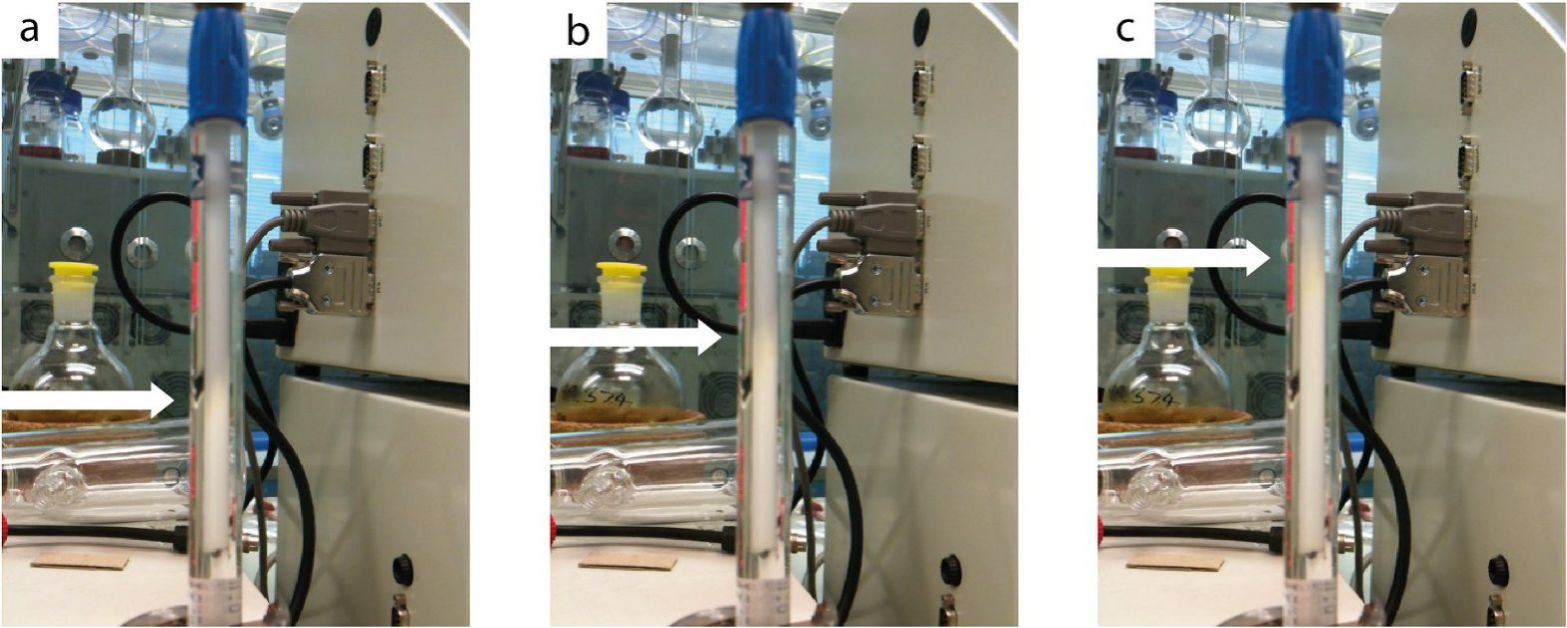
Clips from video footage showing the silica reagent changing appearance; the arrows indicate the edge of the opaque region moving up from the bottom end of the column.

To get around the problem of requiring such a long reaction time, a web-cam was set up to monitor the silica column. The reaction could then be initiated at the end of the day and left to run overnight. During the evening, the live video stream could be viewed remotely to determine the progress of the reaction (Figure 25). If the opaque region had reached the end of the column or if it did not appear to be moving following successive views then the reactor was stopped by issuing a remote power-off command to the heater and pump. A further command to request the status of these devices gave confirmation that powering-off had occurred. This system allowed lab chemistry to be performed when the lab would otherwise be inaccessible (outside normal working hours when lone-working restrictions are in effect) whilst reducing solvent wastage through constant overnight pumping.

### Computer vision augmented automation

**Figure 26:**
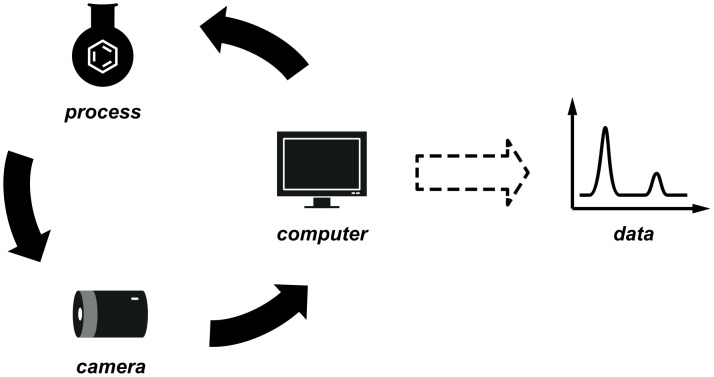
Combination of computer vision and automation to enable machine-assisted synthetic processes.

Digital image recording as described so far can be combined with the automated control of laboratory apparatus for the kind of intelligent interpretation of visual information that would traditionally require the presence of a human operator. Programmatic image processing and computer vision can be used to translate the key content of these images into one or more numerical parameters, which then inform decision-making algorithms to feed back to the reaction control apparatus (Figure 26).

We mentioned earlier an application of video cameras to give an otherwise inaccessible view on the inside of a microwave reactor cavity. A natural extension of this would be for a computer to monitor the video stream and halt the procedure in the case of potentially dangerous events such as microwave arcing. As with the sequestration example above, there are many situations in which such improvements could be made to current procedures.

There are numerous methods reported for the discrimination of substances (for example, solvents) based on their optical diffractive index [88–90]. An example of a robotic component for liquid/liquid extraction as part of an automated synthesis workstation was reported by workers at the Sunitomo Chemical Company [91]. A digital camera is used to distinguish between the phases of two immiscible solutions, which can then be separated.

**Figure 27:**
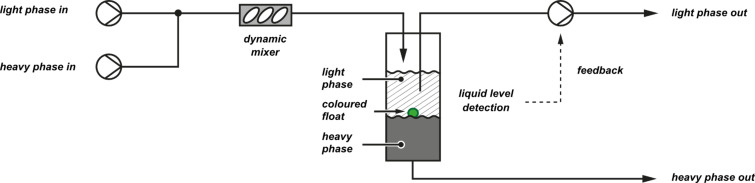
A coloured float at the interface between heavy and light solvents allows a camera to recognise the liquid level. Control of a pump removing the top layer maintains the phase boundary within a controlled region.

Although the exact details of the implementation were not described in this case, our group has recently reported a continuous liquid/liquid extraction system based on a simple visual process [92]. A plastic float with a density intermediate between those of two solvents was placed inside a glass separation column into which the two phases were continuously fed and removed (Figure 27). This increased the visibility of the interface such that tracking was possible by using an image-recognition protocol.

**Figure 28:**
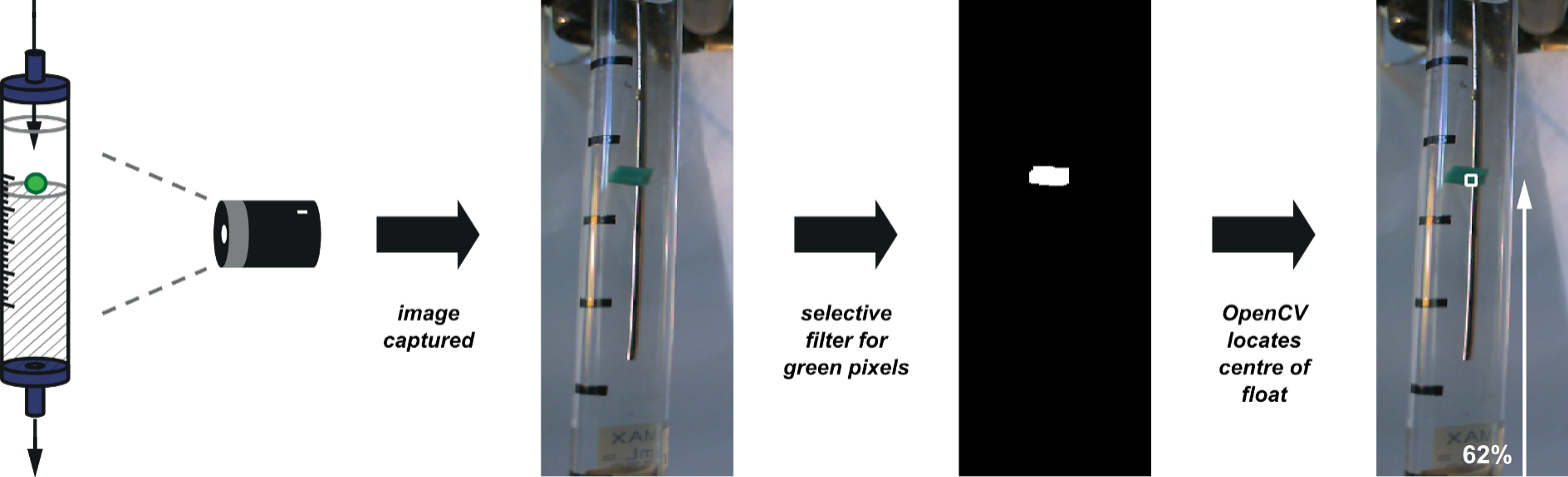
Graphical demonstration of the image-recognition process. At the start of the experiment, the colour of the float is specified. From then on, the programme analyses video frames captured from the webcam by filtering out areas of any other colour and then finds the centroid of the remaining shape, indicated here as a white box. In this frame the float is detected to be at 62% of the height of the image.

Detection was performed programmatically by observing the apparatus with a digital camera and locating the central point of the float with reference to defined points at the top and bottom of the image. A computer programme written in the Python programming language collected images from the digital camera and analysed them using computer vision functions provided by the OpenCV library (Figure 28). In addition to filtration of the image with respect to a colour threshold (green in this case), the processed image was then filtered for noise by using morphological erosion and dilation operations to identify the largest region of colour [93].

Data from each frame were combined to track the motion of the interface in real time. These data were then used directly to adjust the flow rate of a pair of syringe pumps, which removed the aqueous solvent from the top of the separator. With appropriate damping, this functioned as a feedback loop maintaining the interface at the centre of the separator. Integrated control of the syringe pumps within the script was facilitated by the PySerial software library [94], which allows communication via the serial port. This highlights the utility of these open-source resources for the rapid development of new systems.

**Figure 29:**
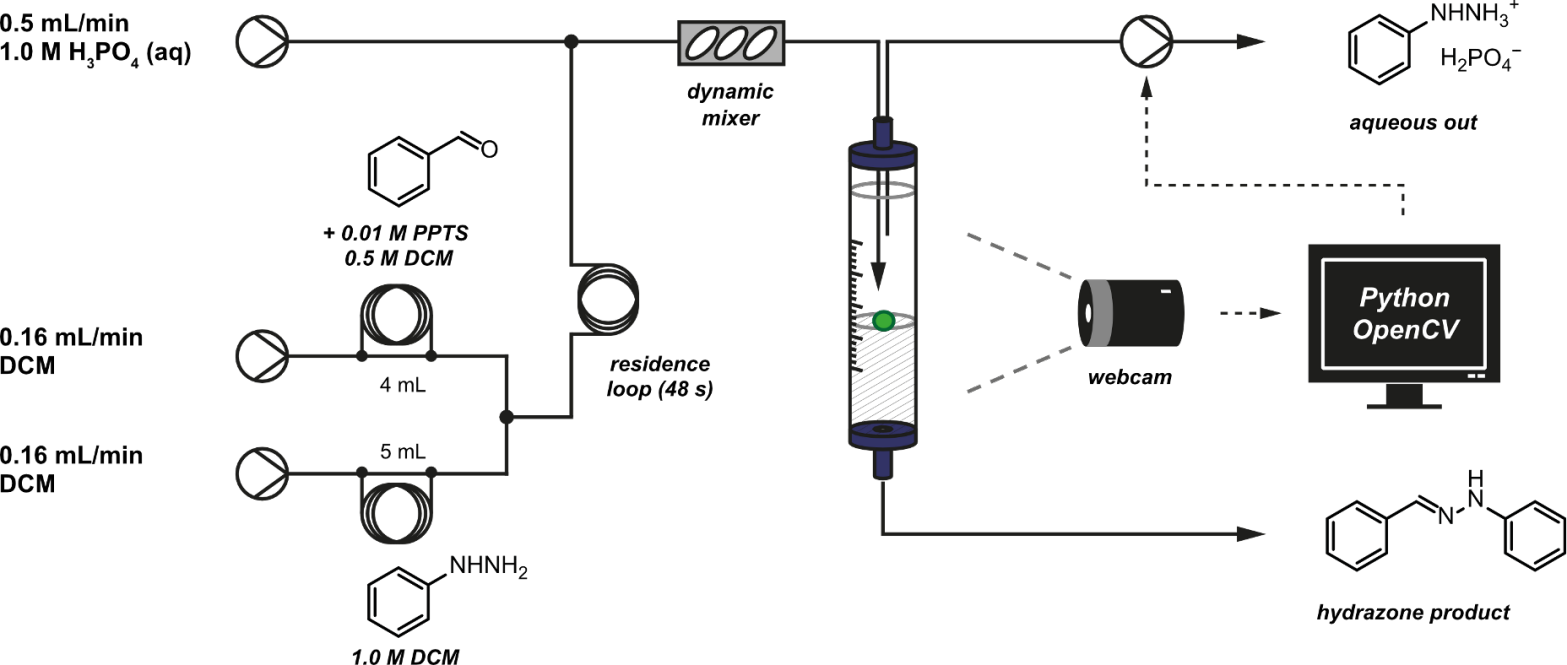
Application of the computer-vision-enabled liquid–liquid extractor. The product mixture of a hydrazone formation reaction undergoes an acid extraction to remove excess hydrazone, and the product is collected in high purity from the organic output.

The resulting steady state allowed continuous liquid–liquid separation for a number of days. The first reported use of this device was for the preparation of a series of hydrazones. A continuous aqueous extraction to remove the excess hydrazine enabled the product to be collected in high purity (Figure 29). Other applications reported in this work include alkene epoxidation and dithiane preparation.

**Figure 30:**
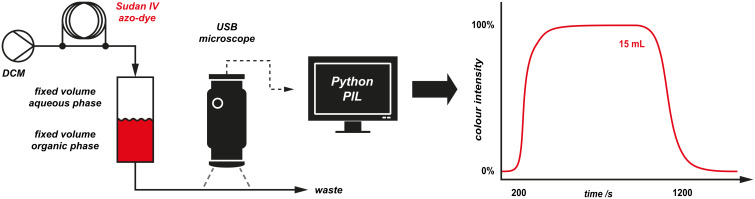
Application of a computer-vision technique to measure the dispersion of a plug of material passing through the continuous-extraction apparatus. The USB microscope camera monitors the outlet flow tube; the intensity of red colour in solution is measured by the software to generate a representation of the dispersion of a plug of dye passing through the separating device.

Dispersion of compounds passing through the continuous extractor was measured by a computer vision technique. The dilution of a red dye injected as a plug was measured by observing the intensity of red colour in the flow tube as observed by a USB microscope camera (Figure 30). Since this dispersion profile should remain fixed for a particular volume of organic phase, these results would facilitate the direct downstream incorporation of another stream of reagent [95], with accurate stoichiometry matching.

During a later evolution of this work, a second generation solvent independent serial liquid–liquid separator was developed to solve extraction problems that occur when partition coefficients are low, for example during diazotization of amino acids in flow leading to the corresponding α-hydroxy acids [96].

**Figure 31:**
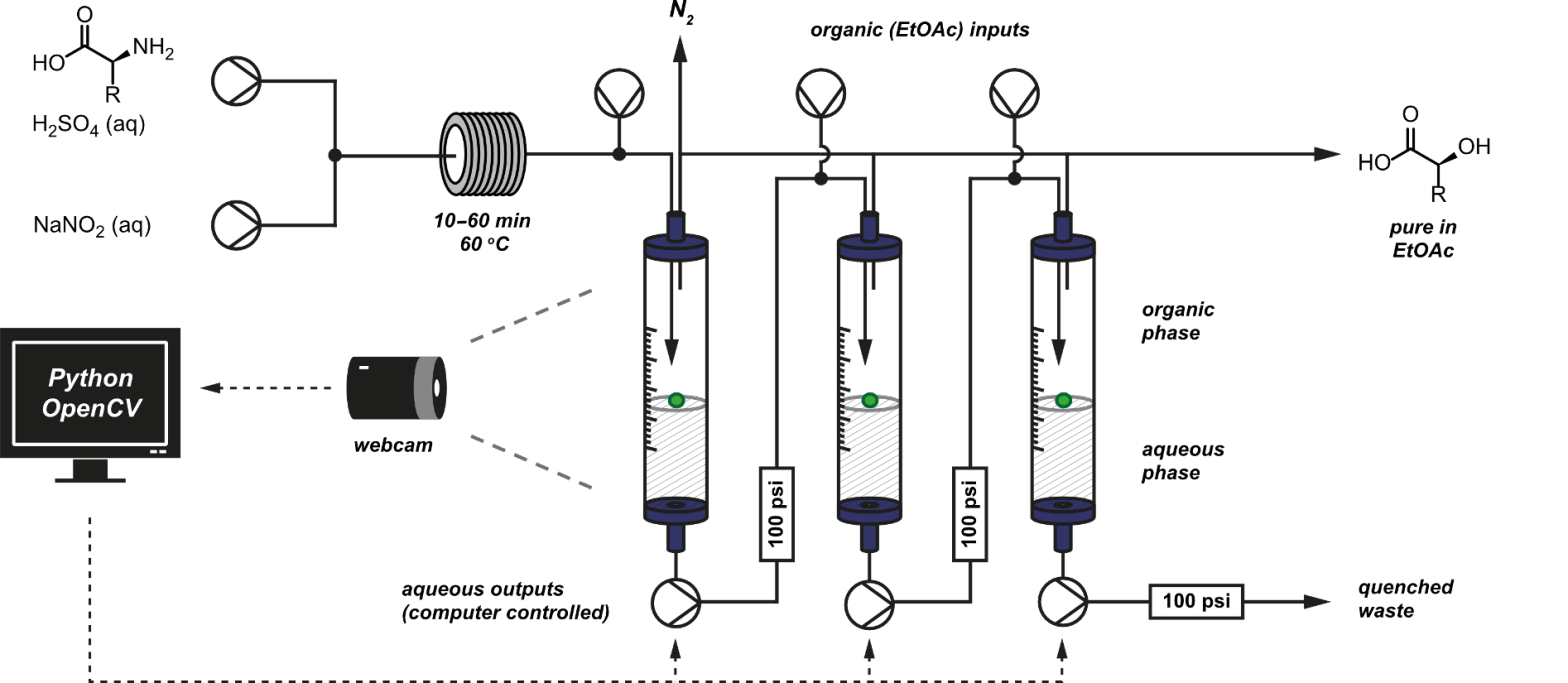
Multiple extractors in series controlled by a single camera.

Three separators were positioned in series to ensure full extraction of the polar product with a variety of solvent mixes. Once again, floating beads at the interface of the layers allowed observation by a digital camera for feedback control (Figure 31). A computer program controlled all of the separators simultaneously, and the complete system remained stable over several 24 hour runs without manual assistance despite turbulence caused by vigorous evolution of nitrogen gas during the diazotization reaction. Batches of over 20 g were readily prepared by operation of the flow system necessitating separation of over 3 L of solvent. In a single run, the full quantity of α-hydroxyisovaleric acid precursor required for the total synthesis of (–)-enniatin B was delivered [97].

Since the extractor device functions with such exact control, no additional organic solvent was added during the separation process. This minimises the amount of solvent used and furthermore allows the output of the extractor to be used directly in another synthetic step. For example, in a recent multistep flow synthesis of branched aldehydes from aryl iodides [98], an in-line aqueous extraction step following an ethylene-Heck reaction allowed the intermediate styrene products to be carried directly into a subsequent downstream hydroformylation reaction. Without the extraction step the hydroformylation step was unsuccessful; experiments suggested that this was due to the amine base or its salts.

**Figure 32:**
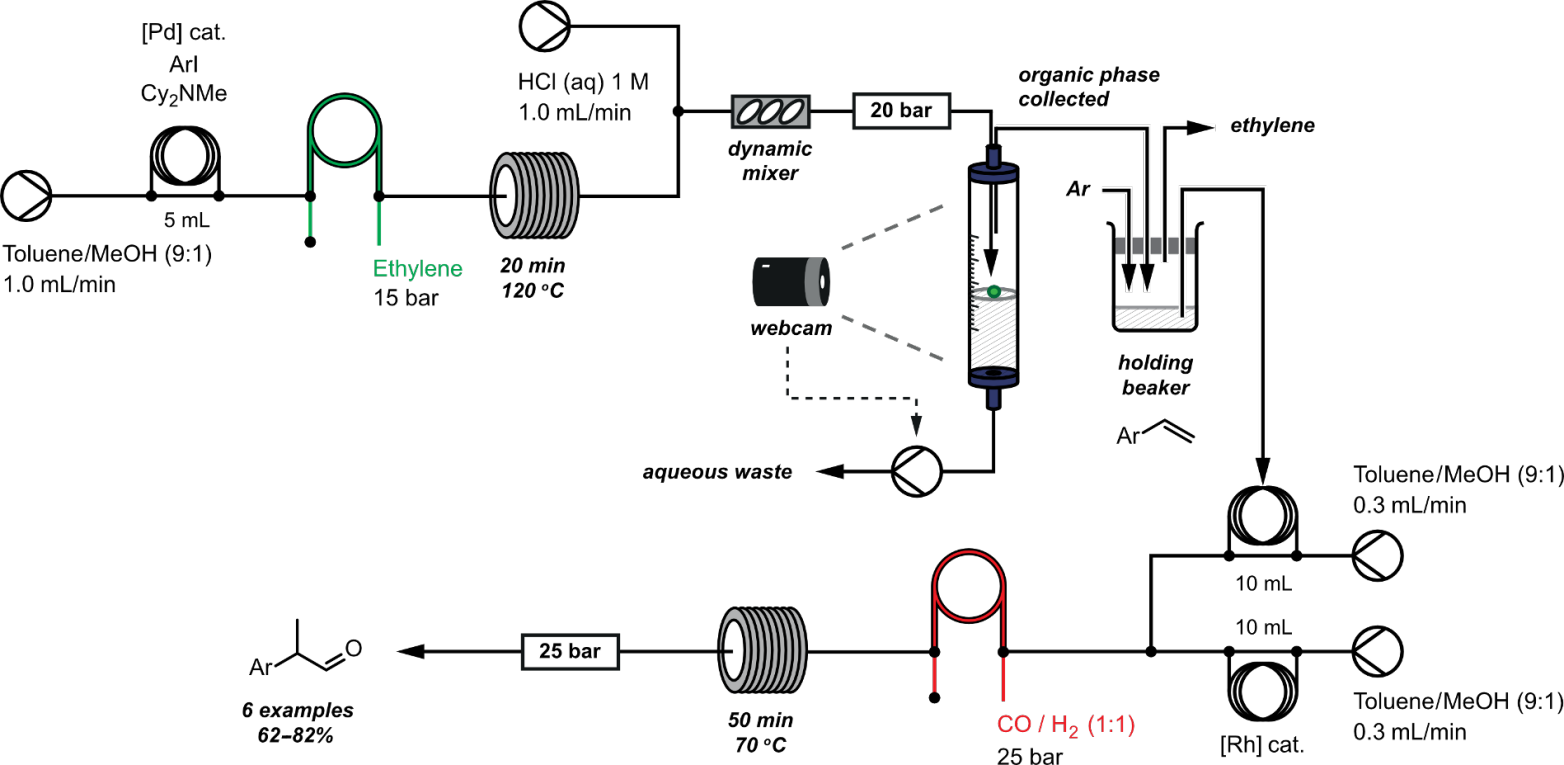
Two-step synthesis of branched aldehydes from aryl iodides using two reactive gases. A liquid–liquid extraction in between the two steps removes the salts and excess base before the second step.

In the reactor configuration used in this work (Figure 32), the output from the ethylene-Heck reaction is combined with an aqueous stream to extract the reaction byproducts. A dynamic mixer promotes complete partitioning of the mixture, before a continuous three-phase extraction is performed by using the camera-enabled apparatus. The organic output is dispensed into a holding beaker from which the residual ethylene can escape under a flow of argon, and from which the solution of products is injected into a second palladium-catalysed gas/liquid reaction. This represents a major step towards a universal and sustainable in-line separation and purification module to enable the direct connection of multiple stages in a flow-synthesis procedure.

## Conclusion

Using the rapidly developing capabilities of computerised digital image capture and visualisation techniques we can envisage that the laboratory of the future will be very different to the one of today. The potential for increased safety, monitoring and control will provide opportunities hardly dreamt of when compared to current practices.

Many of the time- and labour-consuming processes of conventional synthesis programmes are no longer acceptable. Camera-enabled processes can offer viable alternatives in many cases. In addition, we are increasingly called upon to develop more efficient and environmentally benign procedures and to record comprehensive audit trails of our decisions. Consequently, there is a need to provide ourselves with more useful data to inform and record the decisions that we make.

Observation has always informed experimentation and this will certainly continue to be the case; however, today’s access to observation techniques beyond that of the human eye will create new initiatives for more rapid scientific discovery. We believe that the continued introduction of these modern techniques into the laboratory environment will generate increasingly complex methods for monitoring and control, which will be of great benefit to the scientific community. Machine-assisted procedures can benefit from improved reproducibility and more detailed collection and reporting of data throughout a chemical synthesis.

## Supporting Information

File 1 A video assembled from stop-motion photographs of the piperazic acid mixture within the crystallisation apparatus shown in Figure 11. The images were taken at one-minute intervals, and the video was then produced by using each image as a single frame. The crystallisation process can be seen to begin after about 20 s, and is visible against the dark background. A video such as this can be played back once the crystallisation is complete to record the timestamps between which the crystal formation was occurring. A new temperature gradient can then be designed based on these data.

File 2 A video showing a few seconds of footage during the operation of a proof-of-concept magnetic-induced flow mixer [63]. The mixer consists of a polymer tube containing a magnetic stirring bead. Outside the tubing are two electromagnetic coils, which can be energised with opposing voltages from a power supply to attract or repel the stirrer bead. This device was used to enable the processing of heterogeneous slurries in continuous flow.

File 3 A video showing a glass bottle containing a semipermeable polymer tubing, initially filled with an acidic solution of bromocresol green dye. At the start of the video, ammonia gas is flushed through the bottle. The colour change of the indicator dye from orange, to green and then blue shows the pH of the solution increasing as the ammonia gas passes through the tubing and dissolves into solution.
